# Lameness Recognition of Dairy Cows Based on Compensation Behaviour Analysis by Swing and Posture Features from Top View Depth Image

**DOI:** 10.3390/ani15010030

**Published:** 2024-12-26

**Authors:** Ruihong Zhang, Kaixuan Zhao, Jiangtao Ji, Jinjin Wang

**Affiliations:** College of Agricultural Equipment Engineering, Henan University of Science and Technology, Luoyang 471023, China; zhang_rh@stu.haust.edu.cn (R.Z.); 220320261791@stu.haust.edu.cn (J.W.)

**Keywords:** dairy cow, lameness, back key points, motion stability mechanisms, compensatory behaviour

## Abstract

Top-view systems for lameness detection have advantages such as easy installation and minimal impact on farm work. However, the unclear lameness motion characteristics of the back result in lower recognition accuracy for these systems. Therefore, we analysed the compensatory behaviour of cows based on top-view walking videos, extracted compensatory motion features (CMFs), and constructed a model for recognising lameness in cows. By locating the hook, pin, sacrum, and spine positions, the motion trajectories of key points on the back were plotted. Based on motion trajectory analysis of 655 samples (258 sound, 267 mild lameness, and 130 severe lameness), the stability mechanisms of back movement posture were investigated, compensatory behaviours in lame cows were revealed, and methods for extracting CMFs were established, including swing and posture features. The feature correlation among differently scoring samples indicated that early-stage lame cows primarily exhibited compensatory swing, while those with severe lameness showed both compensatory swing and posture. Lameness classification models were constructed using machine learning and threshold discrimination methods, achieving classification accuracies of 81.6% and 83.05%, respectively. The threshold method reached a recall rate of 93.02% for sound cows. The proposed CMFs from back depth images are highly correlated with early lameness, improving the accuracy of top-view lameness detection systems.

## Simple Summary

This study aims to improve the accuracy of top-down lameness detection systems by analysing compensatory behaviours in lame cows. Based on an analysis of the stability mechanisms of back movement posture and compensatory behaviours in lame cows, it is concluded that early-stage lame cows primarily exhibited compensatory swing, while those with severe lameness showed both compensatory swing and posture. Based on compensatory motion features, the lameness classification model achieved an accuracy of 83.05%, with a recall rate of 93.02% for healthy cows. The proposed lameness recognition method assists in the scientific management and automated production of dairy farms.

## 1. Introduction

Milk and dairy products have always been vital sources of dietary energy, protein, and fat for the global population. As a significant livelihood-supporting industry, the dairy sector has long production chains and linkage effects, strongly driving economic development. With the expansion of farm scales and increasing numbers of cows [1], farmers no longer have time to care for each individual animal. Additionally, monitoring cow welfare requires longitudinal measurement of various welfare indicators throughout the cow’s lifecycle [2,3,4]. Therefore, applying modern sensing and computer technologies for the continuous, real-time monitoring and management of cows is crucial for ensuring animal welfare and improving farming efficiency [5,6,7].

Reports indicate that the average lameness rate in cows is 23.5%, causing an annual economic loss of USD 11 billion [6]. Lameness is considered the third leading health issue in dairy cows [8,9,10,11]. It leads to increased direct costs (such as medication, veterinary costs, and mortality) and indirect costs (including reduced milk yield, reproductive performance, and lifespan), significantly impacting cow welfare, health, and farm profitability [12,13,14,15]. While manual lameness scoring is inexpensive, easy to implement, and rapidly adopted, it is inefficient for large herds and subjective, leading to variability between observers [16,17,18]. Automated lameness systems might be more sensitive than traditional methods due to their ability to detect subtle differences and store data over time [19,20].

Lameness changes a cow’s gait [21], manifesting as reduced walking speed, arching of the back, nodding, poor tracking up, and asymmetry [22,23,24,25]. Lame cows often exhibit compensatory behaviours to maintain balance and alleviate hoof pain, which become more pronounced with increased lameness. Automatic detection methods for dairy cow lameness include contact and non-contact approaches. Contact methods use accelerometers or pressure sensors to measure hoof movement [26,27,28,29] and pressure [30,31]. Non-contact measurement methods are primarily based on machine vision technology. They use various imaging devices, including 2D cameras, 3D cameras, and thermal infrared cameras [32], to capture images of cows during their walking process. These images are analysed to assess several gait characteristics such as walking speed, stride length, back arch curvature, tracking up, and hoof temperature [33,34,35,36]. By evaluating these features, these methods can detect the presence and severity of lameness in cows. Non-contact methods are cost-effective, stress-free for the cows, and better suited to large-scale farms compared to contact methods.

Initially, the approach to automatic lameness recognition involved quantifying single or multiple manual lameness scoring indicators. The curvature of the cow’s back contour is a commonly used feature in constructing lameness recognition models [27,37]. By analysing the geometric parameters of the cow’s back contour, the degree of curvature during walking can be described to detect lameness [38,39]. Abnormal gait is a major indicator of lameness in cows. By using image processing to extract the positions of the moving limbs and hooves, drawing movement curves, and analysing these curves, features such as gait asymmetry, speed, tracking ability, relative stride, and ground sensitivity are extracted to build a lameness classification model [33,40,41,42]. Since walking involves the complex interaction of multiple body parts maintaining balance, researchers began to consider the movements of multiple body parts, extracting more detailed and comprehensive indicators than manual lameness scoring [6]. By weighting and combining the motion features of the head and neck, back contour, and all four hooves, a lameness classification model was constructed [22,43,44]. With the advancement of deep learning technology, researchers have started to use convolutional neural networks to extract gait features from cow walking videos, including optical flow maps [45,46], spatiotemporal images of limb movements [47], and skeletal motion images [48], to identify features related to lameness.

The data for the aforementioned studies were sourced from side-view image acquisition systems. Capturing side-view images requires meeting several conditions, including appropriate camera positioning, a stable background, and noise isolation, posing challenges for large-scale application and deployment. Top-view image acquisition systems occupy less space in the environment and have less background noise. Therefore, researchers use cameras placed above the cow walking lanes to obtain walking videos and assess lameness by analysing geometric parameters of the back profile in top-view images [27,49], relative height changes in the left and right hook bones [50], and movement characteristics of the head and neck [51]. Viazzi et al. (2013) localised the spine region of cows from top-down 3D images and obtained the spine curve [52]. They used a least-squares fitting method to fit two ellipses on either side of the highest point R of the spine, extracting four related geometric parameters. A decision tree model was constructed to classify cows into lame and non-lame categories based on these geometric parameters. The authors also extracted spine-fitting parameters from 2D images using the same method and constructed a lameness classification model for comparison. The results showed that the 3D method achieved a recognition accuracy of 90%, providing comparable precision to the 2D side view method, while the top-down view method could overcome limitations related to automation and processing time. Jabbar et al. (2017) used a top-down depth camera to capture depth videos of cows walking. They located the left and right hook bones by analysing the curvature of each point in the depth image [50]. By examining the relative height variation curves of the left and right hook bones in the videos, they classified cows into healthy and lame categories, achieving an accuracy rate of 0.95. These studies demonstrated the potential of top-view depth images in detecting cow lameness. However, the lack of research on the stability mechanisms of back movement poses limitations on the accuracy of top-view lameness detection systems, restricting their large-scale application and deployment.

Top-down depth imaging systems reduce space usage and environmental interference. However, top-down capture systems primarily capture images of the back, and due to the lack of understanding of the stability mechanism of back movements, it is unclear how lameness behaviour manifests in the back. This makes existing lameness recognition methods unable to extract rich features from the movement of back joints, which limits the accuracy improvement of lameness detection systems. Therefore, this study analyses the motion trajectories of key points on the cow’s back, investigates the stability mechanisms of back movement postures, analyses several common compensatory behaviours for lameness, and develops a method for extracting lameness compensation features, thereby creating a lameness assessment model based on back depth videos. This study aims to provide a reference for the construction of a top-down lameness detection system.

## 2. Materials and Methods

### 2.1. Experimental Setup

#### 2.1.1. Data Collection

**Figure 1:**
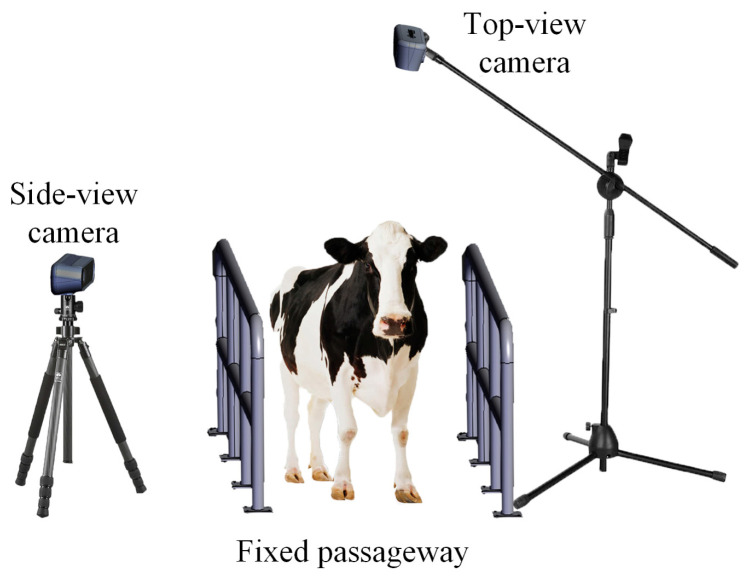
The data acquisition setup.

The experimental data were collected at Shengsheng Farm in Luoyang, Henan Province, China, in October 2023. The subjects were lactating Holstein cows. After milking, the cows walked through a fixed corridor to return to the barn, during which filming took place. A depth camera (Intel RealSense D455, Intel, California, USA) was installed directly above the centre of the corridor at a height of 2.8 meters from the ground to capture the top-view depth videos of the cows. Another RGB camera (Nikon D5200, Nikon, Tokyo, Japan) was placed at the side of the corridor, 5.7 m away from the centre of the corridor and at a height of 1.3 m from the ground, to capture the side-view RGB videos. The top-view video was collected to analyse compensatory behaviour of the back key points. The side-view video was collected to conduct artificial lameness scoring and analyse the interaction mechanism between the back key points and the hind limbs. A schematic diagram of the data acquisition setup is shown in Figure 1. Before capturing the walking videos of the cows, a top view depth image of the background (without cows) was taken for subsequent background removal. When the cow’s trunk was fully within the camera’s field of view, both cameras began capturing simultaneously. When the trunk started to leave the camera’s field of view, both depth cameras stopped capturing simultaneously. The video recording process was manually controlled. The top-view video resolution was 640 × 360, with a frame rate of 30 FPS and a pixel depth of 16 bits. The side-view video resolution was 640 × 360, with a frame rate of 30 FPS. Based on the timestamp information, we adjusted and trimmed the videos captured from the two perspectives to synchronise the cow movements in both videos.

This study collected a total of 860 video segments, and after excluding those containing herd clusters and static frames, 655 segments were retained. Three experienced observers manually scored the videos using a 1–5 scale (1, no lameness; 5, severe lameness) [53]. The most frequent score was used as the final rating, with the median score chosen if discrepancies arose. The scores of the three experienced observers were statistically analysed, and the percentage of agreement (PA) was used to assess the consistency of the scores for the same cow. The formula for calculating PA is as follows:

(1) $$PA=\frac{N_{a}}{N_{all}},$$

where *N_a_* is the number of times the evaluators give the same rating to the same dairy cow, and *N_all_* is the total number of ratings made by evaluators. The summarised rating results showed that for the 5-point scale, the consistency of ratings for samples with a limp score of 1 was 87.5%, for samples with a limp score of 2, it was 81.6%, for samples with a limp score of 3, it was 80.1%, for samples with a limp score of 4, it was 92.9%, and for samples with a limp score of 5, it was 100%.

After manual rating, the dataset included 8 samples rated 1, 250 scored 2, 267 scored 3, 127 scored 4, and 3 scored 5. Considering the small number of samples rated 1 and 5, they were combined with adjacent ratings. According to the 5-point scale, cows with a score of 2, while not exhibiting perfect movement, still had no significant reduction in mobility, so ratings of 1 and 2 were merged into the S (sound) class. Cows with a score of 3, showing some decrease in mobility, were classified into the ML (mild lameness) class. Cows with a score of 4 and 5, which showed significant movement reduction or reluctance to move, were classified into the SL (severe lameness) class. The final dataset consisted of 258 S, 267 ML, and 130 SL samples.

#### 2.1.2. Data Pre-Processing

1. Ground correction

The ground plane was fitted using the Random Sample Consensus (RANSAC) algorithm [54] to obtain the expression of the ground. The normal vector calculated from the ground fitting equation was aligned with the *Z*-axis, and a rotation matrix was applied to rotate the point cloud, causing the ground to become the new XOY plane [55]. In the resulting 3D point cloud of the cow, the walking direction of the cow aligned with the positive *X*-axis, and the direction perpendicular to the ground (pointing upwards) was the positive *Z*-axis. Additionally, in this paper, the left and right sides of the cow were defined from the perspective of observing from the cow’s front. The direction from the left side towards the right side was defined as the positive direction of the *Y*-axis.

2. Background and head–neck region removed

The collected point cloud data contained a lot of irrelevant background noise, such as railings and ground. The first step in data collection was to capture the background point cloud without any cows. We subtracted the Z-values of the background point cloud from the Z-values of the point cloud containing cows according to their index values. We then retained the point cloud regions where the difference was greater than 50 as the cow target point cloud region and set the x, y, and z values of the points with a difference less than or equal to 50 to ‘undefined’. Next, based on the camera parameters, we obtained the resolution of the depth image. We then rearranged the original one-dimensional array of depth values (Z-values) into a two-dimensional array according to the given resolution (number of rows and columns), thereby obtaining the greyscale image of the point cloud. We found the largest connected component in the greyscale image and set the x, y, and z values of all points not belonging to this component to Nan, thereby removing environmental interference. Finally, we filled in the holes in the image. Since this study did not use the head movement of the cow, it was necessary to remove the head and neck region from the depth image. Using statistical methods, the column where the right-side torso area was the narrowest was identified, and the data to the right of that column were removed.

### 2.2. Obtaining the Motion Curve of Key Points

#### 2.2.1. Locating the Key Points on the Back

The hook bone and pin bone of the dairy cows are connected to the four hooves, and their movement is associated with the hooves. When the hooves are diseased, the movement in these areas will also be abnormal, making them key areas for analysing back movement. Research indicates that an arched back is a major sign of lameness in dairy cows [39,52], and thus the spinal line was considered a key movement area. The sacrum, located between the hook bones, reflects the range of movement in the hindquarters and was therefore also chosen as a key movement area. In summary, this study selected the hook bone, pin bone, spinal line, and sacrum as the key movement areas for the back of the dairy cow.

1. Localisation of the spinal line in dairy cows

Based on the physiological structure of dairy cows, the spinal line is located near the symmetrical axis of the cow’s back trunk and is situated at a higher elevation compared to the surrounding back surface. Thus, a method for locating the spinal line was designed as follows:

(1) Initial localisation of the spinal line. A sliding window was used to traverse the entire cow area on the XOY plane. For each window, the sum of the depth values was computed. The window with the maximum depth sum in each column was identified, and the coordinates of the upper-left corner of this window were considered as one of the coordinates for the spinal line.

(2) Fitting the spinal line. The coordinates of the spinal line were fitted using the least squares method. Points that deviated significantly from the fitted line were removed. We extended the spinal fitting line outward in both the positive and negative directions along the *Y*-axis. The region considered as the possible area where the spinal line might exist was denoted as *Br*.

(3) Final localisation of the spinal line. A sliding window was used to traverse the possible region *Br* on the XOY plane. Using a method similar to the initial localisation of the spinal line, we searched for the coordinates of the spinal line again.

2. Localisation of the hook bone in dairy cows

**Figure 2:**
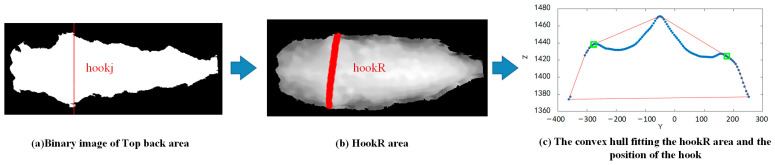
The process of locating the hook bone points. In this figure, *hookj* represents the columns where the hook bone might be located, and *hookR* represents a rectangular region where the hook bone might be located. In (**c**), the two green points indicate the localisation results of the left and right hooks.

The hook bones of dairy cows are paired, protruding skeletal structures symmetrically distributed on either side of the spinal line. Based on their physiological characteristics, the method designed for locating the hook bones was as follows:

(1) Image correction. We rotated the image based on the tilt angle of the spinal fitting line to make the angle of the cow’s spinal fitting line zero.

(2) Locating the top back region. We traversed the cow’s region column by column along the *X*-axis, found the maximum Z-value *Zjmax* for each column, and compared each pixel’s Z-value in that column with *Zjmax*. We removed the pixels where the difference from *Zjmax* was greater than 100. Then, we identified the largest connected component in the image as the top trunk region (*Tb*). Figure 2a provides a binary image of the *Tb* region.

(3) Finding the columns where the hook might be located. We traversed the binary image along the *X*-axis, calculated the sum of pixel values for each column, and identified the column with the maximum pixel sum, which was considered as the potential column for the hook bone, denoted as *hookj*. As shown in Figure 2a, the red-marked columns indicate the potential location of the hook bone, *hookj*.

(4) Locating the hook bone. We defined a rectangular region, *hookR*, centred on column *hookj*. The left and right boundaries of the *hookR* region were defined by extending 10 columns in the negative and positive X-directions from column *hookj*, respectively, while the top and bottom boundaries were set by the upper and lower limits of the trunk. This region was used for locating the hook bone, as illustrated by the red area in Figure 2b.

We ignored the X coordinates of the *hookR* region. To simplify the Y and Z values within the *hookR* region into a single curve, a sliding window was used to traverse the Y−Z scatter plot of the *hookR* region. Each window’s points were replaced by a single point, where the y coordinate of the new point was the mean Y value of all points within the window, and the Z coordinate of the new point was the mean Z value. This process produced a Y–Z fitting curve for the *hookj* column, as shown by the blue curve in Figure 2c.

We constructed the convex hull of the Y–Z fitted curve, which was the smallest convex polygon enclosing the curve, as shown by the red curve in Figure 2c. We then calculated the area between the line segment connecting the i-th and (i + 1)-th points of the convex hull and the Y–Z fitted curve. We identified the two points with the largest areas, which indicated the positions of the two local maximum points at the ends of the Y–Z curve. The two points were used as the left and right hook bone points, *HL* and *HR*, of the cow, as marked in green in Figure 2c.

3. Localisation of the pin bone in dairy cows

**Figure 3:**
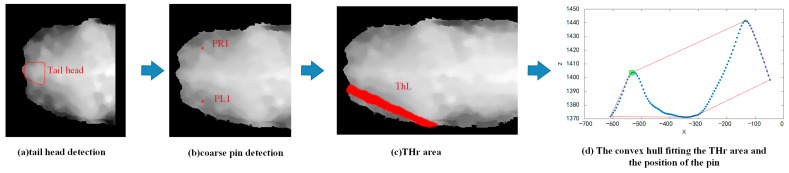
The process of locating the pin bone points. In (**b**), the two red points represent the rough localisation of the pin bones. In (**c**), the red region *ThL* was obtained by extending outward from the line connecting the hook bone and the coarse positioned pin bone. In (**d**), the green point indicates the position of the left pin.

The cow’s pin bones are a pair of prominent, symmetrically arranged structures located on either side of the tail head. Based on their anatomical features, this paper proposed the following method for locating the pin bones:

(1) Tail head localisation method. First, the image was cropped to remove parts where the Y-value exceeded (*hook*j + 40), resulting in the rear half of the cow’s body image. Based on the depth values of this rear half image, the depth image was converted into a colour image. Then, a YOLOv8 [56] model was trained to locate the tail head position within the rear half image. The area within the red contour line in Figure 3a represents the localisation result of the tail head.

(2) Localisation of the pin regions: Based on the tail head localisation results, we defined the left and right pin bone regions in the XOY plane. The columns containing the tail head region were used to determine the columns for the left and right pin regions. The region from the topmost side of the trunk to the maximum row of the tail head was defined as the right pin region (*pinR*), while the region from the minimum row of the tail head to the bottommost side of the trunk was defined as the left pin region (*pinL*).

(3) Rough localisation of the pin bones. The 10 rightmost columns of the left pin bone region (*pinL*) were retained, and the average value of the points in these columns was used as the coordinate value *PL1* for the left hook bone. Similarly, we retained the 10 rightmost columns of the right pin bone region (*pinR*) and used the average value of the points in these columns as the coordinate value *PR1* for the right pin bone.

(4) Precise localisation of the pin bones. The method for precisely locating the left pin bone was similar to that of the right pin bone. A rectangular region, *ThL*, in the XOY plane from the line connecting points *HL* and *PL1* was defined. Its upper and lower boundaries were determined by translating the line upward and downward by 10 pixels. The left boundary was a line perpendicular to the line connecting *HL* and *PL1*, translated 100 pixels in the negative X-direction from *PL1*. The right boundary was a line perpendicular to the line connecting *HL* and *PL1*, translated 100 pixels in the positive X-direction from *HL*. This region, *ThL*, is shown as the red area at the bottom of Figure 3c.

The Y-coordinate of the *ThL* region was ignored. To simplify the X and Z values within this region into a curve, we used a sliding window to traverse the Y–Z scatter plot. We replaced all points within each window with a single point, where the X-coordinate of the replacement point was the average of the X-coordinates of all points within the window, and the Z-coordinate of the replacement point was the average of the Z-coordinates of all points within the window. This process resulted in an X–Z fitting curve, as shown by the blue curve in Figure 3d.

We constructed the convex hull of the X–Z fitting curve, as shown by the red curve in Figure 3d. We calculated the area between the line segment connecting the i-th point and the (i + 1)-th point on the convex hull and the X–Z fitting curve. The point with the maximum area, which determines the local maximum of the X−Z curve’s leftmost extremum, was identified, and we designated this point as the left pin bone point *PL2*, as marked by the green point in Figure 3d. The method for locating the right pin bone point was similar to that of the left pin bone point and will not be repeated here.

4. Localisation of the sacrum in dairy cows

The sacrum is located at the midpoint between the left and right hook bone points. To find its position, we calculated the intersection of the column containing the pin bone points with the spine line. The closest point on the spine line to this intersection was considered to be the location of the sacrum.

#### 2.2.2. Drawing of Motion Curves

Based on the positioning results of the left and right hook bones and the pin bone, we plotted their X–Y and X–Z motion curves and pre-processed the curves. First, we detected and replaced outliers in the curves. Outliers were defined as elements within a specified window length that differ from the local median by more than three times the local median absolute deviation (MAD), a method known as the Hampel filter. This paper used a window length of 5 to detect outliers, and outliers were replaced using linear interpolation based on adjacent non-outlier values. Subsequently, the curve was smoothed using weighted linear least squares and a quadratic polynomial model for local regression, with smoothing performed using 7 data points.

By analysing the linkage between the movement of the cow’s hind hoof and the key points on its back, it was found that the first local minimum in the X–Y movement curve of the cow’s hook typically corresponds to a state where the hind hoof is off the ground, referred to as the “suspension point”. The height of the spine line at the frame corresponding to this suspension point was obtained and used to plot the curve of the spine height at suspension points. Since the absolute height values of the spine were relatively large and may complicate subsequent data processing, these values were converted to relative heights. First, the minimum value of the spine height curve in the first half of the data was identified, and then the difference between each height value and this minimum value was calculated to obtain the relative height curve. Outliers in the spine profile line were then addressed through outlier replacement.

### 2.3. Analysis of the Stability Mechanism of the Key Points

#### 2.3.1. Analysis of Back Compensation Behaviour

To investigate the stability of back movement during cow locomotion, it is essential first to analyse the movement patterns of the back. Direct observation of back movement is challenging. Therefore, this study used simultaneously captured top-view and side-view depth videos to analyse the coordinated movement between the cow’s hind hooves and key points on its back. It revealed the interaction mechanisms and movement patterns between the back and the hind hooves and determined the corresponding moments in the back’s key point motion trajectory for footfalls, lift-offs, stance phase, and swing phase during the cow’s gait cycle.

Next, we analysed the back key point motion trajectories of healthy cows, mildly lame cows, and severely lame cows. By referencing the linkage mechanisms between the back and the hooves and common lameness gait characteristics, we observed abnormal movements and postures of the back key points during limb functional impairment to reveal the mechanisms of back posture movement stability.

#### 2.3.2. Extraction of CMFs

This paper extracted 8 features from the motion curves of the hook, pin, and hook bones, as well as from the spinal height curves. The CMFs were categorised into two types: swing features and posture features.

- The swing features are as follows:

(1) *P_vx_*: The average movement speed of the left and right hook and pin bones along the *X*-axis. The calculation method was as follows: the movement speed along the *X*-axis for the left and right hook and pin bones of the cow was calculated separately, and then the average value was found. This feature reflects the cow’s ability to move forward freely.

(2) P*_vz_*: The average movement speed of the left and right hook and pin bones along the *Z*-axis. The calculation method was as follows: the movement speed along the *Z*-axis for the left and right hook and pin bones of the cow was calculated separately, and then the average value was found. This feature reflects the flexibility of the cow’s joints.

(3) *P_pz_*: The velocity of the pin bone at the moment the hind hoof lands on the ground. The calculation method was as follows: all local minima in the X–Z curve of the pin bone were located. For each local minimum point, we calculated the average change in Z-values within a range of two points on either side of the local minimum. This can be expressed by Formula (2)

(2) $$Ppz=\frac{1}{n}\sum_{i=1}^{n}∆z_{i},$$

where $∆z_{i}$ is the change in Z-values within the specified range around each local minimum point, and *n* is the number of local minima considered.

Based on the analysis of lateral and top-view videos of cows walking, it was found that the local minima in the X–Z movement curve of the pin bone typically correspond to the landing points of the cow’s rear hooves. Therefore, this feature represents the landing speed of the rear hooves and reflects whether they have normal support capabilities.

(4) *P_lr_*: Asymmetry of the pin bones. The calculation method was as follows: the number of points where the Z-values of the left and right pin bones exhibit different trends of increase or decrease was determined. This feature reflects the symmetry of the cow’s movement.

(5) *P_sz_*: Sacrum height change at rear hoof landing. The calculation method for *P_sz_* was as follows: we retrieved the Z-values of the pin bone for the frame corresponding to the rear hoof landing, as well as for the two frames before and two frames after this landing frame. We calculated the difference between the maximum and minimum values in this curve.

- The posture features are as follows:

(1) *P_bm_*: Maximum height of the spine profile at the suspended point. The calculation method for *P_bm_* was as follows: the local minima in the X–Y curves of the left and right pin bones of the cow typically correspond to the moment when the cow’s hoof moves below the body and is in a suspended state. This moment is referred to as the suspended point. We obtained the spine relative height curve at this suspended point and calculated its maximum value, *P_bm_*. This feature value reflects whether the cow has an arched back.

(2) *P_bx_*: The slope of the fitted line for the spine profile at the suspended point. To calculate *P_bx_*, we determined the least squares fitting slope of the spine relative height curve at the suspended point. This feature reflects whether the cow’s body centre of gravity is shifting forward or backward.

(3) *P_d_*: The height difference between the hook and pin bones. To calculate *P_d_*, we computed the difference between the average range of Z-values for the hook bones and the average range of Z-values for the pin bones. This feature reflects the degree of joint flexion in the upper joints of the hind limbs.

#### 2.3.3. Analysis of Feature Correlation Across Different Samples

To determine whether cows with different degrees of lameness exhibit different distributions of swing and posture features, both hypothesis testing and visualisation methods were used to analyse each feature separately.

Distribution tests and location tests were employed to analyse the correlation of compensatory features between different samples. Distribution tests assess if sample data come from populations with a specific distribution, while location tests determine if data come from populations with specific means or medians. This paper used the two-sample Kolmogorov–Smirnov test to check if the compensatory features of different scoring samples had the same distribution and the two-sample t-test to examine if the compensatory features of different scoring samples had the same location value. The results are represented by *p*-values, which indicate the probability of observing a test statistic as extreme or more extreme than the observed value under the null hypothesis. A small *p*-value suggests rejecting the null hypothesis, indicating significant differences between samples; otherwise, the null hypothesis cannot be rejected, implying no significant difference between samples.

A gap feature boxplot was used to visually analyse the correlation of compensatory features between different samples. In a boxplot, the top edge (*T*) and the bottom edge (*B*) of the gap region correspond to Formulas (3) and (4), respectively,

(3) $$T=m+(1.57\cdot IQR)/\sqrt{n},$$

(4) $$B=m-(1.57\cdot IQR)/\sqrt{n}.$$

Here, *m* is the median, *IQR* is the interquartile range, and *n* is the number of data points. At a 5% significance level, boxplots with non-overlapping gaps indicate different medians. The significance level is based on the assumption of a normal distribution.

### 2.4. Classification

Based on CMFs, including swing features and pose features, the dairy cow lameness classification model was constructed. This paper tested two classification methods: machine learning-based classification and threshold discrimination.

#### 2.4.1. Machine Learning

The dataset was divided into training, validation, and test sets at a 6:2:2 ratio, and a 5-fold cross-validation method was used to train the gait classification models. A total of 7 different machine learning algorithms were trained, including the decision tree (DT) algorithm, quadratic discriminant analysis (QDA), logistic regression (LR) classification, the support vector machine (SVM) algorithm, the k-nearest neighbours (KNN) classifier, the ensemble classifier (EC), and the neural network (NN) classifier.

#### 2.4.2. Threshold Discrimination

The concept of the threshold discrimination method involves using a set threshold and the superposition of compensatory behaviour to provide a lameness score for cows. First, suitable thresholds were determined for each feature based on the feature boxplots to assess whether the cow exhibited lameness behaviour corresponding to that feature. Then, the degree of lameness in the cow was evaluated by the accumulation of different lameness behaviours. The steps of the abovementioned method are as follows:

1. Identification of compensatory behaviours

First, a threshold was set for each feature to determine whether the cow exhibited the corresponding compensatory behaviour. For each compensatory swing feature, the threshold was determined using the boxplots of the S class and ML class. If the feature value was negatively correlated with the degree of lameness, the threshold was calculated as the average of the lower quartile of the S class feature box and the upper quartile of the ML class feature box. If the feature value was positively correlated with lameness, the threshold was calculated as the average of the upper quartile of the S class feature box and the lower quartile of the ML class feature box. For each posture feature, the threshold was similarly determined using the boxplots of the ML class and SL class. Next, based on the threshold for each feature and its correlation with lameness severity, we determined if the cow exhibited the corresponding compensatory motion behaviour. A binary value of “0” (normal) or “1” (lameness) was assigned to the feature. Using the described method, the thresholds for the other 8 features could be determined. Subsequently, binary values for these features could be assigned to obtain the two-dimensional features: *B_vx_, B_vz_, B_pz_, B_lr_, B_sz_, B_bm_, B_bx_,* and *B_d_*.

2. Classification of lameness severity

We calculated the features of compensatory swing characteristics *B_mov_* as follows:

(5) $$B_{mov}=B_{vx}+B_{vz}+B_{pz}+B_{lr}+B_{sz}.$$

We calculated the features of compensatory posture characteristics *B_pos_* as follows:

(6) $$B_{pos}=B_{bm}+B_{bx}+B_{d}.$$

Based on the above two compensatory feature values, we used Formula (7) to calculate the lameness score for dairy cows,

(7) $$score=\left\{\begin{array}{cc} S, & \;B_{mov}<4\cap B_{pos}<2\\ ML, & \;B_{mov}\geq 4\cap B_{pos}<2,B_{mov}<4\cap B_{pos}\geq 2\\ SL, & \;B_{mov}\geq 4\cap B_{pos}\geq 2 \end{array}.\right.$$

## 3. Results

### 3.1. Back Key Point Localisation Results

**Figure 4:**
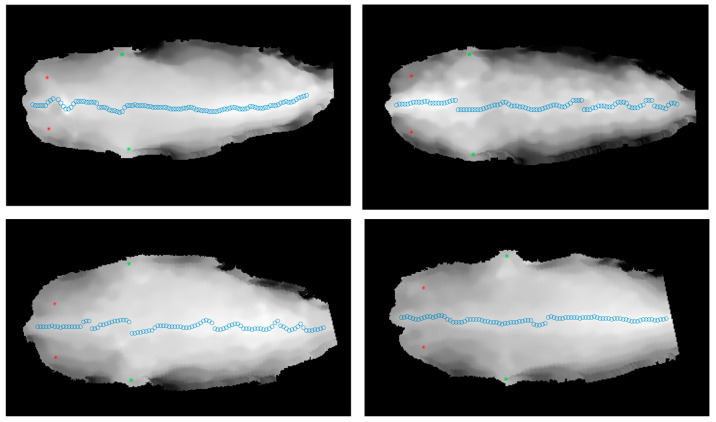
Partial localisation results of the back key points. In this figure, red points represent the localisation results of the pin bone, green points represent the hook bone, and blue hollow circles represent the localisation results of the spine.

This paper evaluated the performance of the back key point localisation model using localisation error (LE). LE refers to the Euclidean distance between the detected coordinates of the target and the true coordinates of the target. A total of 50 frames of images from 50 randomly selected cows from the dataset were used to calculate the average LE of the main key points. Table 1 shows the average localisation error for the main key points, with results indicating that the average error for these key points is relatively small, ranging from 20 to 28 pixels. Figure 4 shows partial localisation results of the back key points. The localisation results indicate that the proposed algorithm exhibits good robustness and can accurately detect the positions of key points for cows of different body types and walking states.

However, in some cases, there were still noticeable localisation errors. The impact of these erroneous points on feature extraction can be significantly reduced or completely eliminated through outlier detection and curve smoothing.

### 3.2. The Stability Mechanisms of the Key Points

#### 3.2.1. Back–Hind Hoof Coordination Mechanism

1. Hind Hoof–Hook Coordination Mechanism

When a cow is standing, its weight is evenly distributed across its four legs. When walking, the weight is supported by one front leg and one rear leg diagonally opposite each other, while the other diagonal pair of legs lift and move forward. The alternating movement of these diagonal pairs of legs causes the cow to move forward. In a healthy cow, a single elevation and lowering of the height of the left and right hook bones correspond to the lifting, forward movement, and landing of the hind legs. The height changes in the left and right hook bones are generally consistent but not perfectly synchronised.

**Figure 5:**
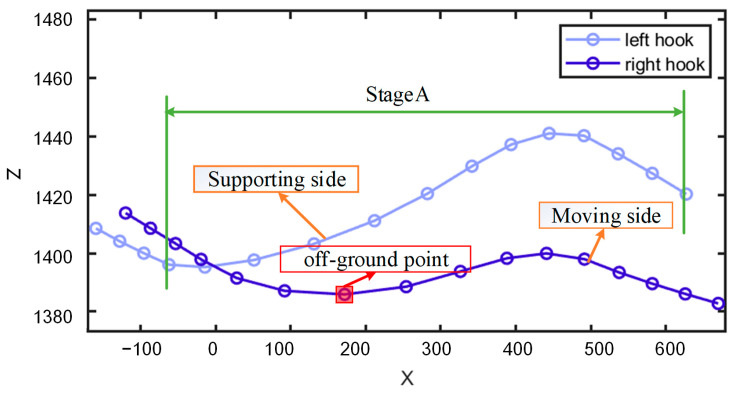
X–Z movement trajectories of the left and right hook bones.

When a cow’s rear hoof on one side begins to move forward, the hook bone on the supporting side will elevate before the hook bone on the moving side. As the rear hoof is lifted and moves forward, both hook bones reach their peak almost simultaneously. As the rear hoof descends, the height of both hook bones decreases simultaneously. This stage of the movement trajectory is shown in Stage A of Figure 5. Then, as the rear hoof on the opposite side begins to move forward, the hook bones on both sides start to elevate sequentially, entering the next cycle of movement. Additionally, Figure 5 also shows that the average height of the hook bone on the moving side is lower than that on the supporting side. Furthermore, the first minimal value point appearing after the intersection of the X–Z movement trajectories of the hook bones usually corresponds to the take-off point of the cow’s rear hoof, as indicated by the point within the red rectangle in Figure 5.

**Figure 6:**
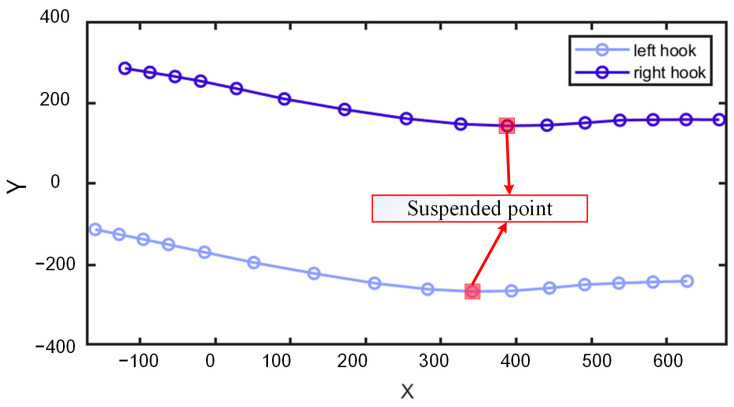
X–Y movement trajectories of the left and right hook bones.

The movement trajectory of the hook bones in the X–Y plane of a cow shows continuous rotation of the body to the left and right while moving forward. The moments corresponding to the extrema in the hook bone’s X–Y movement trajectory are when the cow’s rear hoof is either moving forward beneath the body or about to land, referred to as the “suspension point”. The points within the red rectangle in Figure 6 represent these suspension points.

2. Hind hoof–pin coordination mechanism

**Figure 7:**
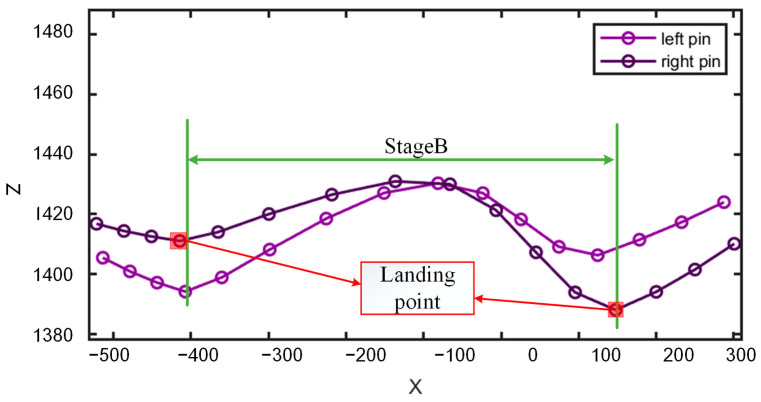
X–Z movement trajectories of the left and right pin bones.

For a healthy cow, the height changes in the rear hoof are almost synchronised with those of the left and right pin bones. One complete cycle of the rear hoof’s lift, forward step, and landing corresponds to a similar cycle of height increase and decrease in the pin bones, as shown in Stage B of Figure 7. The movement trends of the left and right pin bones are generally consistent, with minimal differences in height between their trajectories. Additionally, the local minimum points of the X–Z motion trajectory of the pin on the movement side typically correspond to the landing points of the cow’s hind hooves, as indicated by the points within the red rectangle in Figure 7.

**Figure 8:**
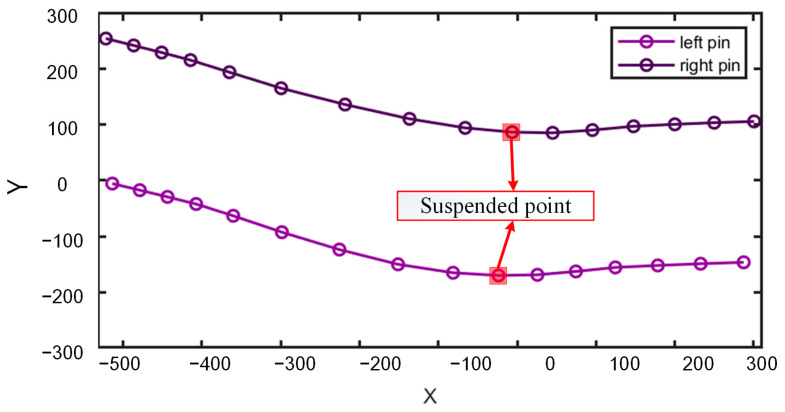
X–Y movement trajectories of the left and right pin bones.

The movement trajectory of the hook bones in the X–Y plane of a cow is similar to that of the hook bones, as the pin continuously rotates to the left and right while moving forward. Similarly, the extrema in the X–Y movement trajectory of the hook bones correspond to moments when the rear hoof is moving forward beneath the body or about to land, known as suspension points. The points within the red rectangle in Figure 8 represent these suspension points.

3. Hind hoof–sacrum coordination mechanism

**Figure 9:**
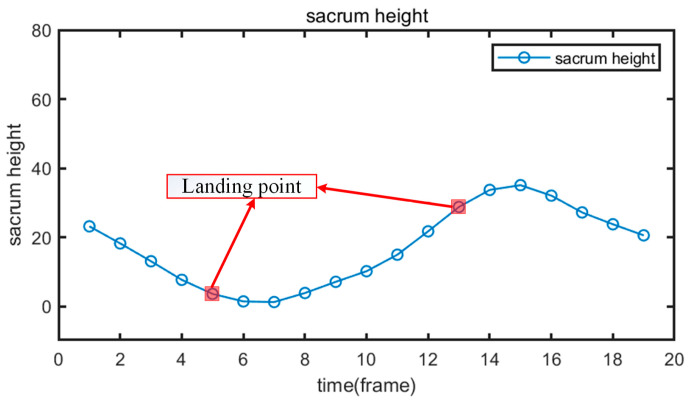
The relative height variation curve of the sacrum.

The relative height variation curve of the sacrum is depicted in Figure 9. When the cow moves forward, the height of the sacrum rises and falls in conjunction with the movement of the rear hoof. The points within the red rectangle in the figure represent the landing points of the rear hoof, as determined from the pin bone movement curve. It is evident from Figure 9 that near these landing points, the sacrum of a healthy cow experiences significant height changes.

#### 3.2.2. Compensation Behaviour

The dairy cow moves forward using a series of repetitive limb movements while maintaining support stability. When this stable movement is disrupted, key points on the back exhibit abnormal movement behaviours and postures to compensate for the movement dysfunction caused by hoof disease. Specifically, this compensation is reflected in both abnormal swing and postures. The abnormal swing behaviours include the following five types:

1. The cow’s ability to move freely decreases, and the speed of forward movement of the hook and pin bones slows down.
2. The cow’s joints become stiff, and the average speed of lifting and landing of the hook and pin decreases.
3. Due to the reduced support capability of the affected limb, the speed of movement of the pin decreases at the moment the hind hoof lands.
4. The upward and downward movement trends of the left and right pins are no longer completely synchronised, increasing asymmetry in movement. During normal walking, the left and right pins should move up or down simultaneously. When one of the cow’s hooves is affected by disease, the cow compensates by altering its movement, causing different trends in the left and right pins.
5. The amplitude of the sacrum up-and-down swing at the moment of hind hoof landing decreases. The sacrum is almost at the centre of the cow’s hindquarters, and its height variation reflects the height changes in the entire hindquarters. During normal walking, the stride and lift height of the hind hoof are larger, resulting in greater up-and-down swing amplitude of the hindquarters and sacrum at the moment of hoof landing. When the cow suffers from lameness, leading to stiff limbs, the stride and lift height of the hind hoof are smaller, resulting in reduced up-and-down swing amplitude of the hindquarters and sacrum. This characteristic indirectly reflects the cow’s movement consistency and stride length.

Abnormal postures include the following three types:

1. The contour of the spinal line is no longer flat, showing a certain degree of curvature. This change is more pronounced during the suspension phase of the hind hoof.
2. Due to the decreased support capability of the affected limb, the body’s centre of gravity shifts either forward or backward. This change is more noticeable during the suspension phase of the hind hoof.
3. The average height of the pin’s movement is significantly lower than that of the hook’s movement. According to the skeletal structure of the cow, the pin is normally slightly lower than the hook. When the cow suffers from severe lameness, the hind hoof’s upper joints flex to lower the centre of gravity of the hind part of the body to alleviate the load. Therefore, in cases of severe lameness, the height of the pin during movement is markedly lower than that of the hook.

#### 3.2.3. Correlation of Features Across Different Samples

1. Hypothesis Testing

To verify the occurrence of the aforementioned compensatory swing and postures in lame cows, distribution tests and location tests were conducted on features from samples with different lameness scores. The results of the distribution tests are shown in Table 2, and the results of the location tests are shown in Table 3. Each *p*-value in the tables is rounded to the third decimal place.

A small *p*-value suggests that the null hypothesis should be rejected, indicating significant differences between samples. From the results of the distribution and location tests, the following conclusions can be drawn: (1) There are significant differences in the features of forward speed (*P_vx_*), lift and landing speed (*P_vz_*), hip joint velocity at landing (*P_pz_*), asymmetry of the hip joint (*P_lr_*), and sacrum movement amplitude at landing (*P_sz_*) between sound and mildly lame cows. However, no significant differences were observed between mildly lame and severely lame cows in these features. (2) The degree of spinal curvature (*P_bm_*) shows different distributions and locations among sound, mildly lame, and severely lame cows. (3) There are significant differences in the centre of gravity shift (*P_bx_*) and joint flexion (*P_d_*) between mildly and severely lame cows, while no notable differences are found between sound and mildly lame cows in these features. (4) All features show different distributions and locations among sound and severely lame cows. The hypothesis testing results indicate that there are significant differences in the swing behaviour between sound cows and mildly lame cows, as well as substantial differences in posture between mildly lame cows and severely lame cows.

2 Visualisation analysis

**Figure 10:**
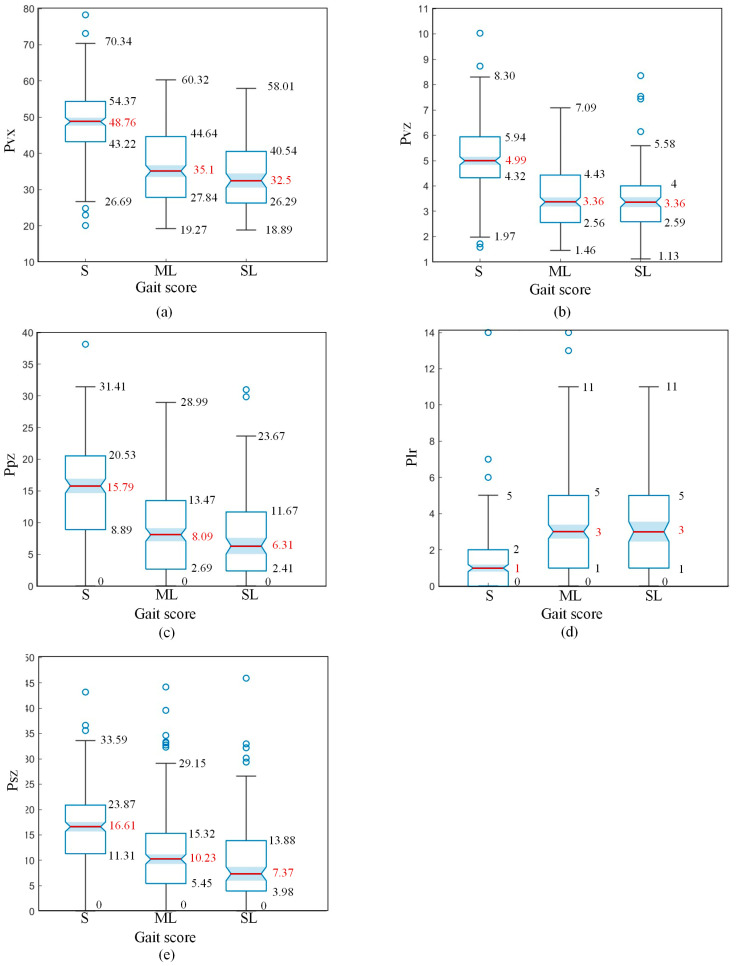
Distribution box plots of swing features for different scores: (**a**) *P_vx_*, the average speed of hook and pin along X-axis. (**b**) *P_vz_*, the average speed of hook and pin along Z-axis. (**c**) *P_pz_*, the velocity of pin at hoof landing. (**d**) *P_lr_*, asymmetry of the pin. (**e**) *P_sz_*, sacrum height change at hoof landing.

**Figure 11:**
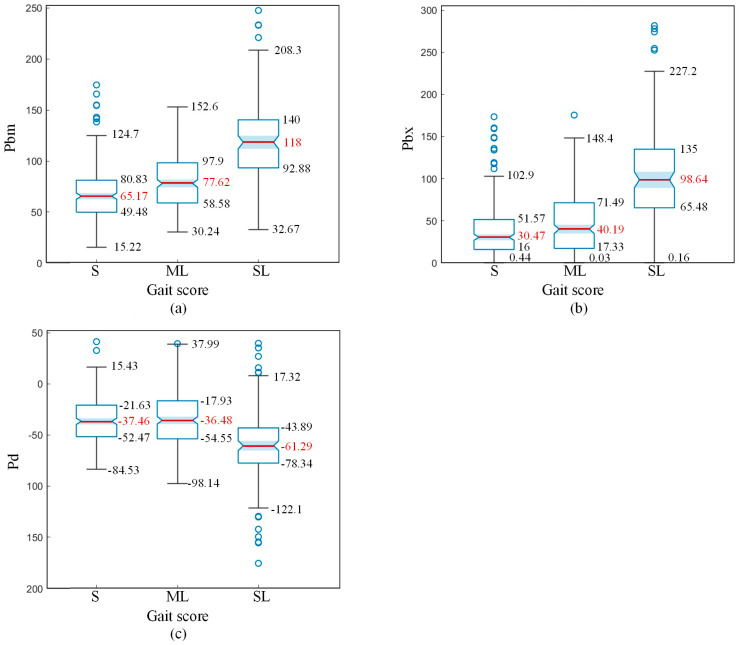
Distribution box plots of posture features for different scores: (**a**) *P_bm_*, maximum height of the spine at the suspended point. (**b**) *P_bx_*, the slope of the fitted line for the spine at the suspended point. (**c**) *P_d_*, the height difference between the hook and pin.

Visualisation analysis transforms complex data into easily understandable formats, thereby enhancing the efficiency and effectiveness of data analysis. Therefore, this study included box plots of compensatory features for differently scoring samples and provided an analysis. Figure 10 shows the distribution box plots of compensatory swing features for different scores. Figure 11 shows the distribution box plots of posture features for different scores.

From Figure 10a,b, it is evident that sound cows (S class) have faster speeds of the hook and pin bones along both the *X*-axis and *Z*-axis while walking compared to mildly lame (ML class) and severely lame cows (SL class). This indicates that lameness reduces a cow’s mobility, causing stiffness in the limbs and restricting full extension during movement. This change can be captured by the average speed of the forward and upward movement of key points on the back. The median values of *P_vx_* and *P_vz_* features for ML and SL class cows are similar, indicating that the mobility of ML and SL class cows is similarly impaired.

According to Figure 10c, the median landing speed (*P_pz_*) of sound cows (S class) is higher than that of mildly lame (ML class) and severely lame (SL class) cows, demonstrating a significant reduction in landing speed for lame cows. This indicates that, at the moment of landing, the supporting ability of the hind hoof decreases due to pain, causing the pin bones to fail to move upward properly. The landing speeds of ML and SL class cows are similar, suggesting that the supporting ability of the hind hoof is equally compromised in both ML and SL class cows.

According to Figure 10d, mildly lame (ML) and severely lame (SL) cows have similar *P_lr_* characteristic distributions, with their medians significantly higher than those of sound (S) cows. This indicates that lame cows exhibit a condition of movement asymmetry.

According to Figure 10e, the amplitude of sacrum movement (along the *Z*-axis) before and after the hind hoof lands is significantly greater in sound (S) cows compared to mildly lame (ML) cows, and mildly lame (ML) cows exhibit slightly greater amplitude than severely lame (SL) cows. This indicates that lameness reduces the amplitude of sacrum movement at the moment of hind hoof landing.

A comprehensive analysis of all backswing characteristics reveals that there is a significant difference in the distribution between sound (S) cows and mildly lame (ML) cows. However, there is a substantial overlap in the distribution of swing features between mildly lame (ML) cows and severely lame (SL) cows. Therefore, swing features can be used as a primary feature to distinguish between sound (S) cows and mildly lame (ML) cows.

As shown in Figure 11a,b, the maximum relative height of the cow’s spine contour (*P_bm_*) and the fitting slope (*P_bx_*) increase with the severity of lameness. The median of *P_bm_* characteristics and the median of *P_bx_* characteristics differ significantly between mildly lame (ML) and severely lame (SL) cows, while the median difference between mildly lame (ML) and sound (S) cows is smaller. This indicates that as lameness becomes more severe, cows begin to exhibit a pronounced arching of the back.

As shown in Figure 11c, the median values of *P_d_* characteristics for sound (S) and mildly lame (ML) cows are −36.68 and −37.46, respectively, which are quite close. However, severely lame (SL) cows have a median *P_d_* value of −61.29, approximately twice that of sound cows. According to the skeletal structure of cows, the height of the pine bone is significantly lower than that of the hook bone, indicating that the hind limbs are in an upper joint flexion state. This suggests that severely lame cows use upper joint flexion to lower their body’s centre of gravity, thereby reducing the weight that the affected hoof needs to bear.

By analysing the box plots of back compensation posture features, it can be observed that the distribution of posture features for sound (S) cows and mildly lame (ML) cows is quite similar, showing much less difference compared to the variation between mildly lame (ML) and severely lame (SL) cows. Therefore, posture features can be used as a primary characteristic to distinguish between mildly lame (ML) and severely lame (SL) cows.

Through the analysis of back compensatory swing and posture features, the following conclusions can be drawn. Early-stage lame cows primarily exhibit compensatory swing, while those with severe lameness show both compensatory swing and posture.

### 3.3. Classification Results

The classification results of the machine learning method are shown in Table 4. It can be seen from this table that the model with the highest classification accuracy is the logistic regression model, which achieved an accuracy of 81.6%. Additionally, the recall rate for healthy (S class) samples was 92.1%.

**Figure 12:**
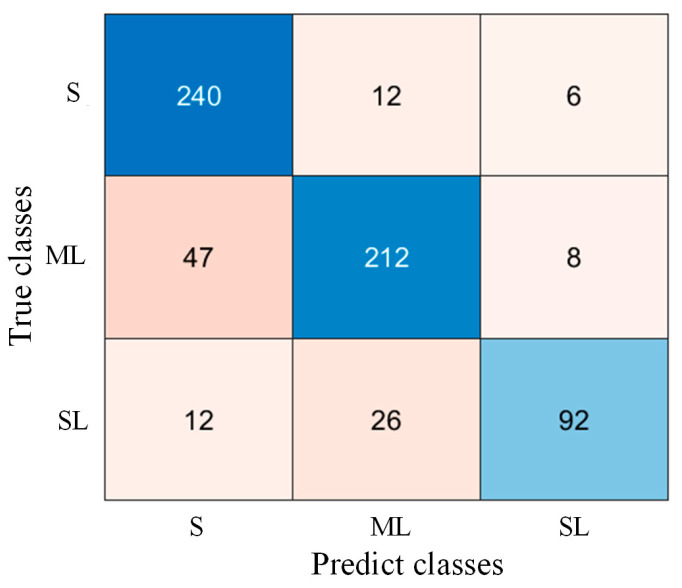
Confusion matrix for the threshold discrimination results.

The accuracy of the threshold discrimination method is 83.05%, with a recall rate of 93.02% for sound (S class) samples, achieving precision similar to that of the side-view lameness detection system. This indicates that the threshold discrimination method can accurately identify sound cows. Figure 12 shows the confusion matrix for the classification results. The confusion matrix reveals that misclassified samples often have adjacent scores.

## 4. Discussion

### 4.1. Compensatory Behaviour and Initial Diagnosis of the Affected Limb

Lameness can be caused by a highly complex array of factors, primarily involving the limbs and adjacent tissues, particularly pain or mechanical disorders in the bones, joints, muscles, nerves, ligaments, tendons, bursae, and hooves. These factors can lead to abnormal movement patterns and postures while walking or standing, reflecting external manifestations of diseases affecting certain limb tissues. Based on the characteristics of lameness, it is classified into support lameness, suspended lameness, mixed lameness, and special lameness, named based on a unique clinical presentation. Suspended lameness is characterised by noticeable dysfunction during the spatial suspension phase. Support lameness is identified by dysfunction during the support phase. When both types are present simultaneously, it is referred to as mixed lameness. Different types of lameness usually correspond to different compensatory behaviours and are associated with different disease locations.

When a cow exhibits compensatory behaviours such as a reduced vertical movement speed of the hook and pin bones (*P_vz_*), a reduced forward movement speed of the hook and pin bones (*P_vx_*), and a smaller amplitude of sacrum sway at the moment of hind hoof landing (*P_sz_*), this indicates that the cow is unable to lift the affected limb high or step far, resulting in slow and uncoordinated movement. These behaviours correspond to suspension lameness, a type of locomotion dysfunction typically caused by diseases affecting the upper limb joints, muscles, fascia, and nerve plexuses.

When a cow exhibits the compensatory behaviours such as a decrease in the pin bone movement speed (*P_pz_*) at the moment of landing, an increase in the asymmetry of pelvic bone movement (*P_lr_*), and an increase in the degree of joint flexion (*P_d_*) in the supporting hind limb, this indicates that the cow is experiencing pain or functional impairment in the affected limb at the moment of landing and weight bearing. These behaviours correspond to a dysfunction in the support phase of movement, known as supporting lameness. This condition is typically caused by diseases affecting the lower limb joints, tendons, ligaments, and hooves.

When a cow exhibits compensatory behaviours indicative of both suspension lameness and supporting lameness simultaneously, the following observations are noted: (1) In the suspension phase, there is a decrease in the movement speed of the key points on the back and a reduction in the swing amplitude. (2) In the support phase, there is a decrease in the landing speed of the key points on the back, an increase in joint flexion, and an increase in asymmetry. This combination of behaviours is classified as mixed lameness. Clinically, isolated suspension lameness and supporting lameness are relatively rare. More commonly, one encounters supporting mixed lameness, which is primarily characterised by weight-bearing issues, and suspension mixed lameness, which is primarily characterised by movement issues. Mixed lameness is often associated with joint diseases in the upper limbs, fractures in the upper limbs, nerve paralysis, or simultaneous conditions affecting both the upper and lower parts of the limbs. Analysing the severity of the two types of lameness compensatory behaviours helps significantly in locating the disease site.

When the slope value (*P_bx_*) of the fitted line for the maximum height of the cow’s spine (*P_bm_*) increases, this indicates that the cow’s centre of gravity is starting to shift from the affected limb to the healthy limb. If the centre of gravity shifts forward, this suggests that there is a problem with the hind limbs. Conversely, if the centre of gravity shifts backward, this indicates an issue with the forelimbs.

### 4.2. Characteristics of the Spine at the Suspended Point

**Figure 13:**
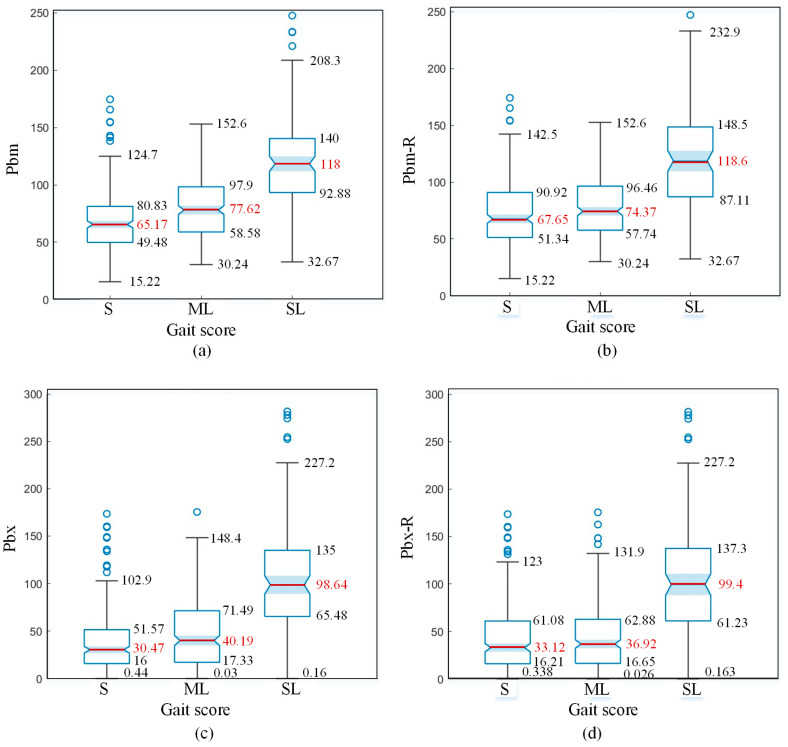
Characteristics of the spine line at the suspended point versus in a random state: (**a**) *P_bm_*, maximum height of the spine at suspended point. (**b**) *P_bm_-R*, maximum height of the spine at random selected point. (**c**) *P_bx_*, the slope of the fitted line for the spine at suspended point. (**d**) *P_bx_-R*, the slope of the fitted line for the spine at random selected point.

Figure 13 compares the box plots of *P_bm_* and *P_bx_* features extracted from the spine line at the suspended point with those of *P_bm_*-*R* and *P_bx_*-*R* features extracted from randomly selected spine lines. Figure 13a shows the box plot of the *P_bm_* feature of the spine line profile in a static state, representing the maximum height distribution of the spine line when a cow’s single hind hoof is suspended. Figure 13b shows the box plot of the *P_bm_*-*R* feature, with each sample’s spine line profile coming from the first frame of walking videos, making the temporal state random. From Figure 13a,b, it can be observed that the median difference between S and ML samples for *P_bm_* features is 12.45, while for *P_bm_*-*R* features, it is 6.74. Therefore, the *P_bm_* feature can better distinguish between S and ML samples compared to the *P_bm_*-*R* feature. There is not much difference in the distribution of *P_bm_* and *P_bm_*-*R* features between ML and SL samples.

Figure 13c shows the box plot of the *P_bx_* feature of the spine line profile at the suspended point, representing the slope magnitude of the spine line when a cow’s single hind hoof is suspended. Figure 13d presents the box plot of the *P_bx_*-*R* feature of randomly selected spine line profiles, where each sample is from the first frame of walking videos, making the temporal state random. From Figure 13c,d, it can be seen that the median difference between S and ML samples for *P_bx_* features is 9.72, while for *P_bx_*-*R* features, it is 3.8. Thus, *P_bx_* features provide better distinction between S and ML samples compared to *P_bx_*-*R* features. There is not a significant difference in the distribution of *P_bx_* and *P_bx_*-*R* features between ML and SL samples.

Thus, it was concluded that while walking, sound cows may show an arched back due to the lifting and landing of their hooves, affecting the spine line profile at certain moments. In contrast, lame cows consistently exhibit an arched back posture at all times. Features extracted from the spine line at the suspended point are more effective for lameness classification compared to those from randomly selected spine lines.

### 4.3. Factors Affecting Recognition Accuracy

1. The motion trajectory is relatively short. Due to the influence of camera performance, shooting environment, and positioning methods, the motion trajectories obtained in this study typically capture only a single lifting, stepping, and landing phase of one hind hoof. This limitation restricts the amount of motion information we can extract from the trajectories, making it difficult to comprehensively observe abnormal behaviours in cows. For example, we observed that during severe lameness, the average height of the supporting side’s hook bone is lower than that of the moving side’s hook bone. However, due to the limitation in the length of the motion trajectories, it is challenging to determine which side is the supporting side and which is the moving side based on the trajectory alone. Consequently, it is difficult to use this feature to identify severely lame cows. In future research, the shooting environment will be improved, key point positioning methods will be optimise, and more accurate key point locations and longer motion trajectories of cows will be obtained.

2. Limited sample size. The collected data exhibit a class imbalance issue, with a notably lower number of severe lameness samples, being only half the number of healthy and mild lameness samples. This hindered a thorough analysis of severe lameness cows’ back abnormalities, leading to insufficient classification performance of the lameness detection model for severe cases. This might explain why the machine learning method’s classification accuracy is lower than the threshold discrimination method. It is observed that a few severely lame cows exhibited a flat back, hopping gait, and faster walking speed. Their back key point CMFs are very similar to those of healthy cows, making it challenging for the features extracted in this study to correctly identify these cows. Additionally, the threshold for the threshold discrimination method was determined based on a limited sample size, so its applicability to other farms remains uncertain. Therefore, the next step is to expand the sample size by collecting walking videos from multiple dairy farms to increase the number of samples, especially those with severe lameness, to better understand the back abnormalities of lame cows and improve the robustness of the lameness detection model.

### 4.4. Comparison with Similar Studies

Based on previous research on top-view lameness detection methods [27,50,52], we, respectively, constructed lameness recognition models using the maximum height and the fitted slope of the spinal line as features (F_1_) and the height difference in the left and right hook bones as features (F_2_). Both recognition models used the Support Vector Machine (SVM) classification algorithm. The recognition results show that the lameness recognition model based on F_1_ features achieved a classification accuracy of 73.8%.The lameness recognition model based on F_2_ features achieved a classification accuracy of 45.5%. According to the classification results, it can be observed that the classification accuracy of the lameness recognition models constructed using the two features mentioned above is lower than that of the lameness recognition model based on compensatory movement features proposed in this paper.

Compared to previous studies on top-view lameness detection methods, the uniqueness of our study lies in analysing the linkage mechanism between back joint points and limb–hoof interactions, proposing and validating compensatory lameness behaviours of back joint points, integrating various types of motion features for lameness recognition, including swing and posture features, and enhancing the accuracy of top-view lameness detection systems.

## 5. Conclusions

In this study, a positioning method for key points such as the cow’s hook, pin, sacrum, and spine was designed, achieving minimal positioning errors and good robustness. By analysing the movement trajectories of these key points, this study revealed the stability mechanism of back movement posture and constructed methods for extracting CMFs. Based on the inter-feature correlation coefficients and feature boxplot distributions, it can be concluded that cows exhibit distinct features at different lameness stages. In the early stages of lameness, cows use compensatory swing to maintain body stability. In severe lameness, both compensatory swing and posture are present to offset the functionality deficits of the affected hoof. Lameness classification was performed using machine learning and thresholding methods, with the machine learning method achieving an accuracy of 81.6% and thresholding at 83.05%. Notably, the thresholding method had a recall rate of 93.02% for healthy cows, offering precision comparable to side-view detection systems. The proposed methods provide valuable insights for developing top-view lameness detection systems and the preliminary diagnosis of affected limbs based on compensatory.

## Figures and Tables

**Table 1:** Average LE of the main key points.

Body Parts	Left Hook	Right Hook	Left Pin	Right Pin
Average LE ^1^	20.33	23.16	27.78	24.96

^1^ The unit of average LE is pixels.

**Table 2:** The *p*-value from the distribution test results between different lameness score samples.

Feature	*P_vx_*	*P_vz_*	*P_pz_*	*P_lr_*	*P_sz_*	*P_bm_*	*P_bx_*	*P_d_*
S-ML ^1^	0	0	0	0	0	0	0.001	0.396
ML-SL ^2^	0.054	0.014	0.137	0.309	0.029	0	0	0
S-SL ^3^	0	0	0	0	0	0	0	0

^1^ In Table 2, S-ML represents the test results between sound (S) samples and mild lameness (ML) samples. ^2^ ML-SL represents the test results between mild lameness (ML) samples and severe lameness (SL) samples. ^3^ S-SL represents the test results between sound (S) samples and severe lameness (SL) samples.

**Table 3:** The *p*-value from the location test results between different lameness score samples.

Feature	*P_vx_*	*P_vz_*	*P_pz_*	*P_lr_*	*P_sz_*	*P_bm_*	*P_bx_*	*P_d_*
S-ML	0	0	0	0	0	0	0.001	0.589
ML-SL	0.018	0.133	0.174	0.143	0.089	0	0	0
S-SL	0	0	0	0	0	0	0	0

**Table 4:** Accuracy of lameness classification models using different machine learning algorithms.

Algorithms	DT	QDA	LR	SVM	KNN	EC	NN
Accuracy, %	77.5	78.5	**81.6**	79.6	76.5	80.6	79.6

## Data Availability

The authors do not have permission to share data.
